# Dr. John G. Wayt

**Published:** 1887-03

**Authors:** 


### Obituary.

---

DR. JOHN G. WAYT died in Richmond, Va. February 15th, 1887. He had only been ill for several days and at first no serious results were anticipated. The deceased was in the seventy-seventh year of age, and had spent nearly half a century of that life in Richmond.

He was a native of Charlottesville and came from that place to this city and established himself as a dentist. He at once secured a large and lucrative practice. He was for many years the leading dentist of the city, and amassed a large fortune as the fruit of his skill and popularity.

During the war he sold much property for Confederate money. He also purchased a farm in Charlotte county and for a time resided there. The war swept away much of his wealth, but he still had a competence.

A number of years ago he retired from active business in favor of his son, the lamented young Dr. George Wayt. The death of this promising young man was a blow from which he never fully recovered. Dr. Wayt was twice married. His last wife was a Miss Greenhow, sister of the City Treasurer of Richmond. This lady survives him. Never were a couple more devotedly attached to each other, and their figures on the streets arm in arm engaged in pleasant and loving converse was a familiar sight, and no two were better known.

The deceased had been for many years a consistent member of the Seventh-Street Christian Church. He was one of the best of men, and needs no word of extended eulogy. A loving husband and father, a faithful friend, a good citizen, an honest man and a consistent Christian, no better or more deserved tribute could be written than this.

In *The State* a short time since in a sketch of the original incorporators of the Male Orphan Society, he was mentioned as the last survivor of the entire number. In this as in all good works he was always interested, and was a contributor.

At a meeting of the Members of the Dental Profession of Richmond, the following resolutions were unanimously adopted:

"The Members of the Dental Profession of Richmond have heard with profound sorrow of the death of Dr. John G. Wayt, whom, at an advanced age and with a name universally crowned with honor and respect by the Citizens of this community, in which he so long resided, it has pleased God to remove from this world. For more than forty years Dr. Wayt practiced his profession in this City and a few men is it allotted to attain such excellence in their chosen life work, or such justly merited honor, and still fewer have done so much to advance the interests of Dentistry. Though incessantly occupied by the cares of a large practice, he yet always found time to lend a helping hand and give a cheering word to the younger members of the profession, seeking to guide them by the light of his experience in their struggle along the road, over which he had so successfully passed. Notwithstanding the fact, that during much of his life, he was dependent upon his practice for his own maintenance, his gratuitous services were always cheerfully rendered to any in need of them. In whatever position he served to promote public interest or Christian benevolence he never failed to take a most active part.

*Therefore be it resolved* that in the death of Dr. Wayt, Richmond has lost one of her most valued and public spirited Citizens, and the Dentists their most illustrious exemplar and respected and beloved co-laborer:

*Resolved* that we hereby tender to the bereaved family of the deceased our sincere sympathies, commending them to Him whose ways are past finding out, but "who is too wise to err and too good to be unkind."

*Resolved* that a copy of these resolutions be forwarded to the bereaved widow of the deceased."

---

